# An evaluation of the parents under pressure programme: a study protocol for an RCT into its clinical and cost effectiveness

**DOI:** 10.1186/1745-6215-14-210

**Published:** 2013-07-11

**Authors:** Jane Barlow, Sukhdev Sembi, Frances Gardner, Geraldine Macdonald, Stavros Petrou, Helen Parsons, Paul Harnett, Sharon Dawe

**Affiliations:** 1 Warwick Medical School, University of Warwick, Gibbet Hill Road, Coventry, UK; 2 Department of Social Policy and Intervention, University of Oxford, Barnett House, Oxford, UK; 3 School of Sociology, Social Policy & Social Work, Queen’s University of Belfast, University Road, Belfast, Northern Ireland; 4 School of Psychology, University of Queensland, Brisbane, St Lucia, Australia; 5 Behavioural Basis of Health, Griffith University, Messines Ridge Road, Mt Gravatt, Qld, Australia

**Keywords:** Parenting, Substance misuse, Drug abuse, Alcohol abuse, Infants, Parents under pressure

## Abstract

**Background:**

Many babies in the UK are born to drug-dependent parents, and dependence on psychoactive drugs during the postnatal period is associated with high rates of child maltreatment, with around a quarter of these children being subject to a child protection plan. Parents who are dependent on psychoactive drugs are at risk of a wide range of parenting problems, and studies have found reduced sensitivity and responsiveness to both the infant’s physical and emotional needs. The poor outcomes that are associated with such drug dependency appear to be linked to the multiple difficulties experienced by such parents.

An increase in understanding about the crucial importance of early relationships for infant well-being has led to a focus on the development and delivery of services that are aimed at supporting parenting and parent–infant interactions. The Parents under Pressure (PuP) programme is aimed at supporting parents who are dependent on psychoactive drugs or alcohol by providing them with methods of managing their emotional regulation, and of supporting their new baby’s development. An evaluation of the PuP programme in Australia with parents on methadone maintenance of children aged 3 to 8 years found significant reductions in child abuse potential, rigid parenting attitudes and child behaviour problems.

**Methods/design:**

The study comprises a multicentre randomised controlled trial using a mixed-methods approach to data collection and analysis in order to identify which families are most able to benefit from this intervention.

The study is being conducted in six family centres across the UK, and targets primary caregivers of children less than 2.5 years of age who are substance dependent. Consenting participants are randomly allocated to either the 20-week PuP programme or to standard care.

The primary outcome is child abuse potential, and secondary outcomes include substance use, parental mental health and emotional regulation, parenting stress, and infant/toddler socio-emotional adjustment scale.

**Discussion:**

This is one the first UK studies to examine the effectiveness of a programme targeting the parenting of substance-dependent parents of infants and toddlers, in terms of its effectiveness in improving the parent–infant relationship and reducing the potential for child abuse.

**Trial registration:**

International Standard Randomised Controlled Trial Number Register: ISRCTN47282925

## Background

Drug and alcohol dependency is a significant public health problem with major human, social and economic consequences. In 2009/10, 8.6% of adults in the UK had used one or more illicit drugs within the last year, and 3.1% of adults had used Class A drugs. These rose to 20% and 7%, respectively, in the 16- to 24-year-old age group. During 2009/10, 206,889 people were in contact with structured drug treatment services (those aged 18 and over) [1].

The evidence suggests that around one-third of drug users in the UK are women, of which as many as 90% are of childbearing age [2]. Estimates also show that around 2% to 3% of children under 16 have a parent who is a problematic drug user and around 1% of births are to drug users and a similar number to problem drinkers [3]. Of children less than 1 year old, it has been estimated that 19,500 live with a parent who has used Class A drugs in the last year and 93,500 live with a parent who is a problem drinker [4].

Drug-dependent parents are at high risk for maltreatment of their children. Around 25% of all children subject to a child protection plan are cared for by a parent with a substance misuse problem [5] and one study found a significantly higher risk of child protection proceedings amongst infants of substance-misusing parents compared with infants of non-drug users (32.4% vs. 7.1%) [6]. These figures are similar for other countries such as the US [7,8] and Australia [9].

The first 2 years of a child’s life are a particularly important developmental phase, primarily because of the impact of early parent–infant interaction on the infant’s developing neurological [10] and attachment systems [11]. Substance-dependent parents demonstrate a range of parenting difficulties and deficits [12], and in particular a reduced capacity for sensitivity and attunement during this important period [13-16].

The poor quality of caregiving is influenced by the problems that co-occur with drug dependence, such as, psychiatric disorders and psychopathology [14], particularly disorders of affect regulation [17]. The infants of drug-dependent parents may also have a range of neurobiological problems as a result of drug exposure *in utero*[18,19] making it difficult to assess whether the compromised interaction is due to the impact on the infant’s neurobehavioural system, the dyadic organisation of the interaction or both [15]. These infants are also often exposed to a range of other substances including psychomotor stimulants such as alcohol and nicotine [20].

The research mentioned above highlights the need for interventions that target the parenting of substance-dependent parents in addition to a focus on drug dependency, and four systematic reviews [21-24] have examined the impact of community-based substance-misuse treatment programs combined with interventions aimed explicitly at improving parenting. These show that studies that have focused explicitly on infants or toddlers are limited and include standard home visiting programmes [25-28], which have on the whole showed limited evidence of effectiveness, and a number of more promising approaches including mentalisation-based programs in community [29,30] and residential settings (not discussed further here) [31,32]. Other promising interventions that have been used with substance-dependent parents of older children include standard parenting skills and case-management approaches [33,34], psychotherapeutic relational therapy groups [35,36], court-based models of working (not discussed further here) [37], and the mindfulness-based programme being trialled here [9].

Focus on Families (FOF) [33] was one of the earliest attempts to combine behavioural family therapy and parenting skills training with home-based case management and clinic-based relapse prevention, to support methadone-maintained parents of children aged 3 to 14 years. This approach achieved significant reductions in parental drug use, and improvements in terms of parental skills, deviant peers and family management (*ibid*), and although there were no between-group differences in substance-use disorders at the 15-year follow-up, males in the FOF group had a significantly lower risk of developing a substance-use disorder compared to those receiving standard care [34].

Another approach that has recently been developed to support methadone-maintained mothers of older children is Relational Psychotherapy Mother’s Groups (RPMGs) [35,36], which comprises a 24-week programme involving a supportive therapist, interpersonal relational focus group treatment and insight-oriented parenting skill facilitation. The focus was on psychological functioning in terms of reducing anger, depression and guilt, in addition to addressing specific parenting issues by encouraging the use of alternatives to physical punishment, alongside age-appropriate discipline and warmth. A randomised controlled trial (RCT) comparing RPMGs with recovery training found that despite initial gains in the post treatment phase in terms of self-reported child maltreatment, cocaine abuse, emotional adjustment and depression [35], these results were not maintained to the 6-month follow-up [36]. The authors suggest that this may have been due to the abrupt cessation of the therapy programme.

The concept of mentalisation refers to the capacity to understand the actions of self and others in terms of intentional states (for example, thoughts, beliefs and desires), and has been proposed to be a key mechanism in improving affect regulation and caregiving competence [38], and may be a significant factor in the functioning of high-risk substance-dependent women [32]. This construct has been incorporated into a number of treatment approaches targeting high-risk mother-infant/toddler dyads including maltreating parents of infants [39], high-risk women of infants where there are issues such as trauma and unresolved loss [40] and substance-dependent parents of toddlers [29]. The Mothers and Toddlers Program (MTP) [29] comprised a 12-session weekly ‘individual parenting therapy’ for mothers with a child under 36 months who were enrolled in outpatient substance-use treatment. The intervention incorporated a range of strategies explicitly focused on enhancing maternal capacity for reflective function and reducing distorted mental representations of parenting. The findings of an RCT [29] comparing MTP with a parent education programme, showed that post treatment, mothers in the MTP group had significantly higher scores for ‘self-focused’ but not ‘child-focused’ reflective functioning, that were maintained to the 6-week follow-up [30]. There were also group differences in caregiving behaviour and child behaviour (that is, increased communication and a delayed effect for the contingency score was found at the follow-up), and in levels of depression and psychiatric distress. Substance use improved across both groups. Group differences in maternal caregiving behaviour were maintained to the follow-up, but not maternal depression, and the results for psychiatric symptoms favoured the Parent Education Programme (PE) group. Maternal representations of the child were significant at the follow-up only [30]. These findings suggest that this intervention has considerable promise in the short term when delivered by highly trained and supported therapists.

Overall, a number of innovative ways of working with substance-dependent parents of infants and toddlers have been developed during the past 10 years. Recognising the importance of helping women who are experiencing a range of problems to manage their roles as mothers has been a central theme. This is a significant challenge for the field, and although promising approaches are emerging, the duration of the programmes and focus on the wider social context in terms of social support have been highlighted as key factors requiring careful consideration. The current study adds to this literature by providing a rigorous evaluation of a programme with demonstrated efficacy [9]. Further, the current evaluation involves the programme being delivered by front-line practitioners engaged in routine clinical practice, many of whom do not have formal qualifications in social work or psychology, thereby comprising a real-world effectiveness study.

### Rationale for the parents under pressure programme

Adverse outcomes for the children of drug-dependent parents, including child maltreatment, are not associated specifically with parental drug use as a single risk factor, but rather with the complex interplay between child functioning, parental drug use, parental psychopathology, parenting practices, family environment (including spousal relationships and the availability of social support) and socioeconomic factors such as unemployment and poverty [41].

The relationship between impulsivity and poor affect regulation has been widely documented in the substance-abuse literature and it is clear that these play a role in the aetiology of substance abuse in addition to having an impact on treatment outcome [42]. The seminal work by Linehan in her development of dialectical behaviour therapy emphasised the importance of addressing dysregulated affect in women with a range of impulse control disorders including substance abuse [43-45]. Notably, this work addressed the problem of dysregulated affect through the explicit incorporation of mindfulness-based approaches that aim to increase capacity for self-regulation of attention in terms of a focus on immediate experience, alongside the adoption of a particular stance with regard to that experience, which is characterised by curiosity, openness and acceptance [46,47]. However, evidence on the effectiveness of approaches that include other components, such as case management [33] and social support [29], suggests that treatment needs to address multiple domains of family functioning.

### Parents under pressure programme

The Parents under Pressure Programme (PuP) was developed as an intensive home-based intervention underpinned by an integrated framework of family functioning [48], which draws strongly on attachment theory with a focus on developing a safe and nurturing relationship. The programme is underpinned by a recognition that the quality of the parent–child relationship is related to the parent’s capacity to provide sensitive, responsive and nurturing caregiving [45], and the parent is helped to recognise their own strengths and potential difficulties using video feedback, shared discussion with the practitioner and completion of exercises using the parent’s workbook. Additionally, difficulties in managing dysregulated affect and impulsive behaviour, both in relation to parenting and to substance abuse, are addressed through the use of mindfulness exercises and a focus on recognising and managing negative emotional states. In relation to the former, these include exercises that involve mindfulness meditations in addition to helping a parent develop a greater awareness of being fully present in the moment with their infant during daily activities (for example, taking pleasure in watching an infant sleep, and during bath time and play). In relation to the latter, the use of techniques such as ‘urge surfing’, understanding cravings and learning to manage negative mood states without the use of substances, complement the care received from the standard drug and alcohol treatment services [46,47].

Evaluation of the programme with parents on methadone maintenance of children aged 3 to 8 years found significant reductions in child abuse potential, rigid parenting attitudes and child behaviour problems [9,49]. There was also a significant reduction in methadone dose, within a treatment context in which methadone doses were largely determined by parental choice. Changes in dose therefore reflected client decision-making rather than treatment policy that was abstinence focused [9].

The PuP programme contains 12 modules and these are delivered across 20 calendar weeks. The selection and delivery of the modules are determined by the assessment. The programme is embedded within a case-management framework, and day-to-day issues such as housing and finance provide a therapeutic opportunity to put coping skills into practice in a mindful and emotionally contained manner. Sessions are conducted in the home and last between 1 and 2 hours with content drawn from the parent’s workbook. Additional case management occurs outside the treatment session, according to individual family needs (for example, housing, legal advice or school intervention).

The programme begins with a comprehensive assessment and individual case formulation conducted collaboratively with the family. Specific targets for change are identified during the assessment, which then become the focus of treatment. Each module comprises a theme that continues throughout treatment. For example, Module 6, *Connecting with Your Baby*, focuses on helping a parent connect with their child through a series of exercises that help the parent reflect on their own relational experience with their baby. There is an emphasis on learning their baby’s language, and ‘mindful play’ in which a parent is taught to use mindfulness constructs to observe, describe and participate during play and special times.

Module 7, *Mindful Child Management*, teaches non-punitive child management techniques and locates these within a developmental context to ensure that parents understand the most age-appropriate strategy to use. This also requires a sensitive understanding of the baby’s or child’s cognitive capacity and developmental charts supplement the parent’s workbook as a way of helping parents feel proud about their baby’s or child’s development while also developing realistic views about their baby’s or child’s capacity. Mindfulness techniques are used to help parents gain greater control over their own emotional responsivity in both stressful parenting situations, such as prolonged crying of an infant, and situations requiring behaviour management in order to reduce impulsive, emotion-driven punishment [50,51].

The use of the modules depends on the personal situation of the parent. For example, the *Relationship* module includes a focus on improving communication in intimate relationships. It also includes sections on defining the qualities of a good and loving intimate relationship for couples with a troubled relationship history.

### Study objective

The objective of this study is to evaluate the effectiveness, cost effectiveness and user acceptability of the Parents under Pressure programme for substance-dependent parents of infants less than 2.5 years of age.

#### *Hypotheses*

The delivery of a 20-week mindfulness based intervention to parents with substance-abuse problems will reduce the potential for child abuse. The programme will also improve parent–infant/toddler interaction, infant/toddler social and emotional adjustment, and parental psychological well-being in terms of stress, depression and anxiety, and the capacity for affect regulation. This will also be manifest in reduced substance use. The impact of the programme on the primary outcome (that is, child abuse potential) will be mediated via the parent’s capacity for affect regulation.

## Methods/design

### Study design

The study comprises a mixed-methods, multicentre randomised controlled trial involving an explanatory sequential design, in which qualitative data will be collected in addition to the quantitative effectiveness and cost-effectiveness data, with the aim of providing further understanding of the data that has been obtained.

### Ethical and research governance approval

The study has been granted ethical approval from the Biomedical Research Ethics Committee at the University of Warwick (BREC reference number 189-03-2012).

### Project timescale

The trial commenced in October 2011 and will end in December 2015. Recruitment of participants will be staggered across the six trial sites.

### Randomisation

Randomisation will be stratified by centre using minimisation [52], and consenting parents will be randomly allocated to one of two arms, using a computer-generated numbers table (using Stata v7) by an independent statistician. The intervention arm will receive the Parents under Pressure programme alongside standard services for substance-dependent parents, and the control arm will receive standard services alone, which will vary by site but will mostly be adult facing and linked to the management of addiction and relapse, alongside the implementation of child protection services as appropriate. Standard services will also provide families with access to universal health and parenting support, children’s centre support and self-help groups in addition to the more targeted support from drug and alcohol treatment services.

To reduce bias and contamination, study staff and researchers involved with delivering the PuP intervention will have no contact with families in the control group. All data will be collected by a researcher who is blind to group allocation (see Data-collection process). Some contamination within centres may still occur, however, because there is a small population of families at each site and families in the intervention group may pass ‘helpful’ information to families in the control group. Furthermore, participants may reveal their treatment allocation to the researcher whilst undergoing data-collection activities, such as the qualitative interviews. An assessment of the extent to which contamination has occurred will be made as part of the process of data collection (for example, recording any unmasking of group allocation during interviews with families and asking if families know others in the study). If levels of contamination are found to be high in either study group, an extra confounder variable denoting contaminated controls will be added to the analysis and the effects of this contamination investigated (see Data analysis section). However, we do not expect that contamination between groups will be a significant issue because the ‘dose’ received by control families will be low compared with that received by the intervention group (that is, the intensive 20-week programme).

### Study participants

The study will include individuals who meet all the following criteria:

➢ They are a primary caregiver with responsibility for a child under the age of 2.5 years (if the child is removed from the parents during the study, the intervention will continue on the premise that the child will be returned).

➢ They are receiving treatment for a drug or alcohol problem including opioid replacement treatment, relapse prevention or other treatment programme. If both parents have an alcohol or drugs problem, only the mother will be assessed.

➢ They are able to understand spoken English.

The following individuals will be excluded from the study:

➢ Parents whose child is not residing with them and does not have contact with them at the beginning of the intervention and where there is no plan for reunification.

➢ Pregnant women (unless the baby is due within 4 weeks of the recruitment period) who have no other child under 2.5 years residing with them.

➢ Women in a relationship in which there is active and ongoing domestic abuse or who are actively psychotic or expressing active suicidal ideation.

### Sample size

Dawe and Harnett [9] found an effect size (ES) of 0.92 using the Child Abuse Potential Inventory (CAPI) scores, in an RCT of the PuP programme in Australia with parents of children aged 3 to 8 years. This is a large change and the current study, which is being conducted with a younger population of children (under 2.5 years), should therefore be powered to detect a much smaller change.

Power calculations for the current study show that in the region of 54 women are required in each arm of the study to detect a change in the region of 0.5 ES with 80% power and 0.05 significance. Considerably more families (that is, in excess of 147 per arm) would be required to be confident of detecting a change that is smaller than this (that is, 0.45 ES). Allowing for dropout in the region of 5% [9] necessitates that approximately 114 families are recruited to the study (that is, 57 in each arm).

### Recruitment

Recruitment will take place over the course of a year at six participating centres, each of which will be required to recruit around 19 families per year. Assuming an uptake rate of 1:3, each centre will be required to refer five women to the study per month, to achieve a total of 114 participants over a year.

Referrals will be made by any worker who has contact with families who are in a drug or alcohol treatment programme including midwives, drug treatment centre workers, staff at children’s centres and staff working with charitable organisations within the field. They will provide eligible families with a brief information sheet inviting them to receive more information about the study, and asking them to provide their consent to pass on their contact details to the research team. The research team will call the family, inform them that they will receive some information in the post and agree to call them in 5 days to answer any questions, and to hear their decision about taking part. Primary carers who are interested in taking part will then be visited by a researcher at home or a venue of their choice to discuss any queries they may have about participation. Participants who agree to take part will be asked to provide written informed consent by initialling, signing and dating a study consent form, which will be witnessed by the researcher. Written informed consent will always be obtained before any study-specific procedures including collection of baseline data. A telephone randomisation procedure will be instigated, and the participants will be contacted within 24 hours of the visit to inform them about their allocated group.

### Data-collection instruments

#### *Primary outcome measures*

*Child abuse potential* will be assessed using the Brief Child Abuse Potential Inventory (BCAPI) [53], which is a 33-item self-report questionnaire developed to identify individuals at risk for physical child abuse, with an agree/disagree format. BCAPI has a 9-item validity scale and a 24-item abuse risk scale. The internal reliability of the full CAPI abuse scale is high, with KR-20 correlation coefficients ranging from .92 to .96 and good test-retest stability of .91 and .83 for 1-day and 1-month intervals, respectively [53]. BCAPI is highly correlated with the full CAPI [54], and has a good inter-item consistency of .88 [55].

#### *Secondary outcome measures*

*Parent–toddler interaction* will be assessed using the infant and toddler versions of the CARE-Index [56]. The CARE-Index requires a 3-minute video of the parent with their child. It measures three aspects of maternal behaviour (sensitivity, covert and overt hostility, and unresponsiveness) and four aspects of a toddler’s behaviour (cooperativeness, compulsive compliance, difficultness and passivity). Scores range from 0 to 14, higher scores indicating better sensitivity and/or co-operation and so on, and the scores for each are interdependent such that a high score for maternal sensitivity is related to a low score for hostility and unresponsiveness. Similarly a high score for infant co-cooperativeness is related to a low score for difficultness, passivity or compulsive compliance. The CARE-Index has been shown to discriminate abusing, neglecting, problematic and adequate dyads [57]. The inter-rater reliability for the infant CARE-Index was 0.75 or above for four of the seven variables. The inter-rater reliability for maternal sensitivity was 0.81, maternal unresponsiveness 0.87, maternal control 0.85, infant cooperative 0.57, infant compulsive 0.96, infant difficult 0.99 and infant passive 0.98 [57].

The toddler version of the CARE-Index will be used with children over 2.5 years of age. This newly developed version of the tool has a good inter-rater reliability of .93 but a rather low test-retest reliability of .40 [58]. There is, however, a significant correlation between the maternal sensitivity score on the toddler CARE-Index and child attachment security using the Preschool Assessment of Attachment classification (PAA) [58,59].

*Infant social and emotional adjustment* will be assessed using the Brief Infant and Toddler Socio-emotional Adjustment Scale (BITSEA) [60], which comprises a 42-item parent-report measure of infant and toddler (that is, 1- to 3-year-old children) social and emotional adjustment. It comprises two subscales – competence and problems measured using a three-point Likert scale. A higher score for the competence subscale and a lower score for the problems subscale indicate better adjustment. It has an inter-rater reliability ranging from 0.55 to 0.78 [60] and internal consistency of .79 for the problems scale and .65 for the competence scale. It has a test-retest reliability ranging from 0.79 to 0.92, and it also discriminates children with clinically significant problems from matched subjects [60]. 

*Parental psychological functioning* will be assessed using the Depression, Anxiety and Stress Scale (DASS-21), which is a 21-item self-report instrument involving a four-point Likert scale designed to measure the three related negative emotional states of depression, anxiety and tension/stress [61,62]. Cronbach’s alphas for the DASS-21 subscales are .94 for depression, .87 for anxiety and .91 for stress [63]. The measure correlates well with other measures of depression, anxiety and stress [64].

*Parenting stress* will be measured using the Parenting Stress Index short form (PSI-SF) [65], which is a well-validated self-report measure comprising 36 items measured using a five-point Likert scale of perceived stress in the parenting role. PSI-SF scores are highly stable over a 1-year period, based on a subsample of 21 abusive parents. Correlations between the first and second assessments are as follows: .61 for the personal distress scale, .75 for the childrearing stress scale and .75 for the total scale [66].

*Emotional regulation* will be measured using the Difficulties in Emotional Regulation Scale (DERS) [67], which is a 36-item self-report measure of difficulties with emotion regulation. Each item is rated on a five-point Likert-type scale that reflects the proportion of time for which an individual exhibits a particular aspect of emotion regulation; higher scores are indicative of greater difficulties with emotion regulation. The scale gives a score for total difficulties with emotion regulation. The instrument also has good test-retest reliability for a 4- to 8-week period of .88 for the total score, and adequate reliability for the six subscales (.69 for non-acceptance, 0.69 for goals, 0.57 for impulse, .68 for awareness, .89 for strategies and .80 for clarity) [67]. DERS has demonstrated good convergence of validity with established measures of emotional dysregulation, negative emotionality, emotional avoidance, worry, panic and generalised anxiety [67-69]. Additionally, DERS has been shown to adequately predict behavioural outcomes believed to be associated with emotion dysregulation, such as intimate partner abuse, self-harm and aggression [67,70]. The validity of the DERS subscales has been established via multiple factor analytic studies [67,70] and by evidence that the subscales are differentially associated with internalising and externalising behaviour [70].

*Severity of borderline personality* will be assessed using the Personality Assessment Inventory – Borderline (PAI-BOR) [71]. The full Personality Assessment Inventory (PAI) comprises 344 items covering constructs most relevant to a broad-based assessment of mental disorders. PAI-BOR focuses on borderline features and is a 24-item self-report questionnaire using a four-point Likert scale with four non-overlapping subscales that measure the essential features of borderline personality disorder: affect instability (BOR-A), identity problems (BOR-I), negative relationships (BOR-N) and self-harm (BOR-S). These four subscales were designed to measure the unique features given by DSM-IV. PAI-BOR has an internal consistency of .84 and a test-retest reliability of .86 over a 3- to 4-week time period [72-74].

*Parental drug/alcohol use* will be confirmed using case records and measured using Timeline Follow-back (TLFB), a widely used calendar-based method of assessment [75]. Each interview is structured using a calendar in which recent events, such as payday social events, are used as memory aids to assist in recall. This is a reliable and valid measure of substance use; patients’ reports of their drug consumption using this method generally had high a retest reliability exceeding .85, convergent and discriminant validity with other measures, agreement with collateral informants’ reports of patients’ substance use, and with results from patients’ urine assays [76]. The number of days of substance use (including amphetamines, cannabis, alcohol and heroin) in the 30 days prior to assessment will be recorded. This self-report will be validated using hair toxicology in a random sample of cases (10%) [77].

#### *Practitioner evaluation*

*Therapist alliance* will be assessed using the Working Alliance Inventory short form (WAI-SR) [78], which is based on Bordins’ original model of alliance. The full Working Alliance Inventory (WAI) consists of 36 items on a seven-point Likert scale [79]. WAI-SR comprises 24 items – 12 items that measure a therapist’s experience of the client and 12 items measuring the client’s experience of the therapist, each of these focusing on tasks, goals and bonds. The items are scored on a seven point Likert scale with 1 being never and 7 being always. WAI-SR is highly correlated with the full WAI and can serve as an adequate substitute (that is, the bond scales have a correlation of .94 and .91, the goal scales .91 and .86, and the task scales .83 and .87) [78].

*Programme dose, programme integrity and programme compliance* will be assessed using a range of measures designed for this study to capture the duration, frequency and focus of the sessions. Programme integrity will be assessed using the PuP Therapist Experience Measure (TEM) [9], which examines the extent to which the client perceives the key concepts of the programme to have been covered. It is based on the Therapist Adherence Measure developed for use in multi-systematic therapy [80], which found high scores to be predictive of the client outcome.

### Data-collection process

Data will be collected by a researcher who is blind to study arm, in the respondent’s home or at a drug treatment centre. Study participants will be requested not to disclose their group allocation to the researcher, and loss of blinding will be recorded and taken into account at the analysis stage. The data will comprise a range of self-report questionnaires that are robust to loss of blinding, and independent observations of the parent and infant, which will be rated externally to the research team. The researcher will provide assistance with the completion of self-report questionnaires where this is required. Data will be collected at baseline, immediately post-intervention and at the 6-month follow-up.

### Process evaluation

#### *Aim*

The aim of the process evaluation is twofold.

First, it is to identify the extent to which factors such as the amount of intervention received and practitioner evaluation affect the impact of the intervention. This will involve exploratory statistical analyses to examine the inter-relationships between the individual contexts of both families and centres, and the treatment mechanisms for change embedded within the programme in terms of their impact (that is, the outcomes obtained).

Second, it is to enhance understanding of the user and provider experiences of the new service. A range of data will be collected from participants who refuse to take part in the study or drop out of the intervention or study; this will include quantitative demographic data and qualitative data to explore the reasons for non-participation or discontinuation. In-depth interviews will also be conducted with: a) practitioners and supervisors following the training and during the delivery of the intervention, b) a range of stakeholders (for example, partner agencies, local commissioners and national opinion leaders) and c) a purposive sample of participating parents. The latter will be selected using quantitative outcome data to identify study participants who show change and those who show no change, and the aim of the interviews will be to gain a better understanding of the factors that contributed to these outcomes.

### Data-collection methods

Candidates for interview will be invited to take part in an interview by letter, and will be provided with an information sheet explaining why they have been invited to take part and the wider study context and a consent form. Interviews will be conducted at a time and location convenient to the stakeholder. Where necessary, telephone interviews will be conducted. With the permission of the interviewee, all interviews will be recorded.

The interviews will be conducted using a semi-structured interview schedule, which will provide a list of key topics to be explored. Interviews with stakeholders will focus on the following: the experience and adequacy of the training and ongoing supervision, issues relating to referral and embedding of the PuP service within the wider service context and the perceived benefits and difficulties relating to the delivery of the service. Interviews with the purposive sample of service recipients will focus on the following: perceptions about the PuP service and provider, aspects of the services that were experienced as favourable or unfavourable, ways in which the service helped or hindered recovery, experience of other services and so on.

### Data management and analysis

Interview data will be fully transcribed and coded using the qualitative data analysis package NVivo 8. Thematic analysis will be undertaken to identify the key themes that emerge across the data. A narrative summary of the key themes identified will be presented using quotations selected on the basis of their capacity to demonstrate some aspect of the identified theme.

### Economic evaluation

A prospective economic evaluation, conducted from a NHS and personal social services perspective, will be integrated into the trial. The economic assessment method will, as far as possible, adhere to the recommendations of the NICE Reference Case [81]. Primary research methods will be followed to estimate the costs of delivering the PuP programme, including development and training of accredited providers, the cost of delivering the intervention, participant monitoring activities, and any follow-up or management. Broader resource utilisation will be captured through two principal sources: (i) participant questionnaires, adapted from the Client Services Receipt Inventory, administered at each follow-up point; and (ii) data from routine data-collection systems. Unit costs for health and social care resources will largely be derived from local and national sources and estimated in line with best practice. Primary research using established accounting methods may also be required to estimate unit costs. Costs will be standardised to current prices where possible. Cost-effectiveness analysis will be undertaken on two main outcome measures: parent–infant interaction (CARE-Index) and child abuse potential (CAPI) at 6 months.

Results will be presented using incremental cost-effectiveness ratios and cost-effectiveness acceptability curves generated via non-parametric bootstrapping. This accommodates sampling (or stochastic) uncertainty and varying levels of willingness to pay for reductions in the primary outcomes of interest. Additionally, net benefit statistics will be estimated. A series of sensitivity analyses will explore the effects of uncertainty surrounding key parameters on the incremental cost-effectiveness ratios. One such analysis will adopt a societal perspective incorporating costs to other sectors of the economy, direct costs to trial participants and their partners, informal care provided by family and friends, and productivity losses.

### Data analysis

All data will be analysed using an intent-to-treat analysis, unless significant deviations from the intended treatment arm are detected, in which case a per protocol analysis may also be conducted. Descriptive statistical summaries (for example, medians and ranges, means and variances, or contingency tables, depending on the variable distribution and type) will be presented for the primary and secondary outcome measures at each time point. Baseline data will be scrutinised to check comparability between treatment arms and to highlight any characteristic differences between the six trial recruitment sites. Any disparities found will be added to subsequent models to compensate for these differences.

Data collected from families who were approached but refused to take part and those who dropped out will also be analysed and examined for trends (using only basic information such as consenting participant age, gender and intervention arm), alongside qualitative data concerning the reasons for dropping out. We will also investigate potential contamination between intervention groups, as described in the Randomisation section above.

#### *Primary analysis*

Changes in the CAPI score between baseline and at the 6-month follow-up will be compared for the intervention groups using ANCOVA with the site where the family was recruited used as an independent confounder.

#### *Subsidiary analyses*

In a similar manner to the primary outcome measures, changes in the secondary outcome measures at baseline and at the 6-month follow-up will also be compared for the intervention groups using ANCOVA. We will also examine the differences at baseline and the immediate post-intervention follow-up period for all outcome measures.

To further investigate the effects of confounding variables, subject to the limitations of the data, we will use multi-level linear mixed modelling between intervention arms. The use of a combination of random and fixed effects, will enable us to model the impact of factors such as therapist, site, family composition and family trajectory.

We will also construct regression models to investigate the relation between the primary outcome measures (that is, CAPI) and other potential mediating variables (that is, measures of parental mindfulness and affect regulation), to address the hypothesis that the impact of the programme on the primary outcome will be mediated via the parent’s capacity for affect regulation.

Multiple imputation (MI) will be used to compensate for missing data at different assessment points. Imputation assumptions for MI will be reported and justified, and imputed data analysed as part of a sensitivity analysis.

Qualitative data will be fully transcribed and analysed thematically using NVivo 8. The limitations of the qualitative data will be assessed in terms of its credibility, transferability, dependability and confirmability [82]. This data will also be examined on a case-by-case basis in terms of the mixed-methods analysis, to better understand the reasons for the success, or otherwise, of the intervention.

## Discussion

Over the last decade there has been an increasing interest in the development of interventions that address the multiple needs of substance-dependent parents, particularly in terms of reducing the impact that their dependency issues have on developing infants and toddlers. We found four reviews that examined innovative ways of supporting the parenting of this group of parents, and these suggest that there are still few programmes that explicitly focus on supporting parents of children less than 2 years of age.

The Parents under Pressure programme has been shown to be effective in reducing the potential for child abuse in children ranging from 3 to 8 years of age. This study will provide evidence for the clinical and cost effectiveness of an adapted Parents under Pressure programme with children under the age of 2.5 years. The data will be used not only to explore the impact of the intervention on both child abuse potential and parent–infant/toddler interaction, but the factors that appear to mediate its impact, alongside parents’ perceptions of the reasons for success or otherwise. The findings will be examined alongside evidence from other studies that have examined new approaches to supporting substance-dependent parents of very young children.

## Trial status

The study is in the process of starting recruitment.

## Abbreviations

BCAPI: Brief child abuse potential inventory; CAPI: Child abuse potential inventory; DASS-21: Depression, anxiety and stress scale; DERS: Difficulties in emotional regulation scale; ES: Effect size; FOF: Focus on families; MI: Multiple imputation; MTP: Mothers and toddlers program; PAI-BOR: Personality assessment inventory – borderline; PSI-SF: Parenting stress index short form; PuP: Parents under pressure; RCT: Randomised controlled trial; RPMG: Relational psychotherapy mother’s group; PE: Parent education program; WAI-SR: Working alliance inventory short form.

## Competing interests

SD and PH developed the Parents under Pressure programme, and they deliver training programmes internationally.

## Authors’ contributions

All authors contributed to the drafting of the final and approved version of this manuscript.
